# Supplementary figures and images for: Gut microbiota dysbiosis and neurological function recovery after intracerebral hemorrhage: an analysis of clinical samples
Source: Microbiol Spectr. 2024 Sep 24;12(11):e01178-24. doi: 10.1128/spectrum.01178-24 (PMC11537008; doi:10.1128/spectrum.01178-24)

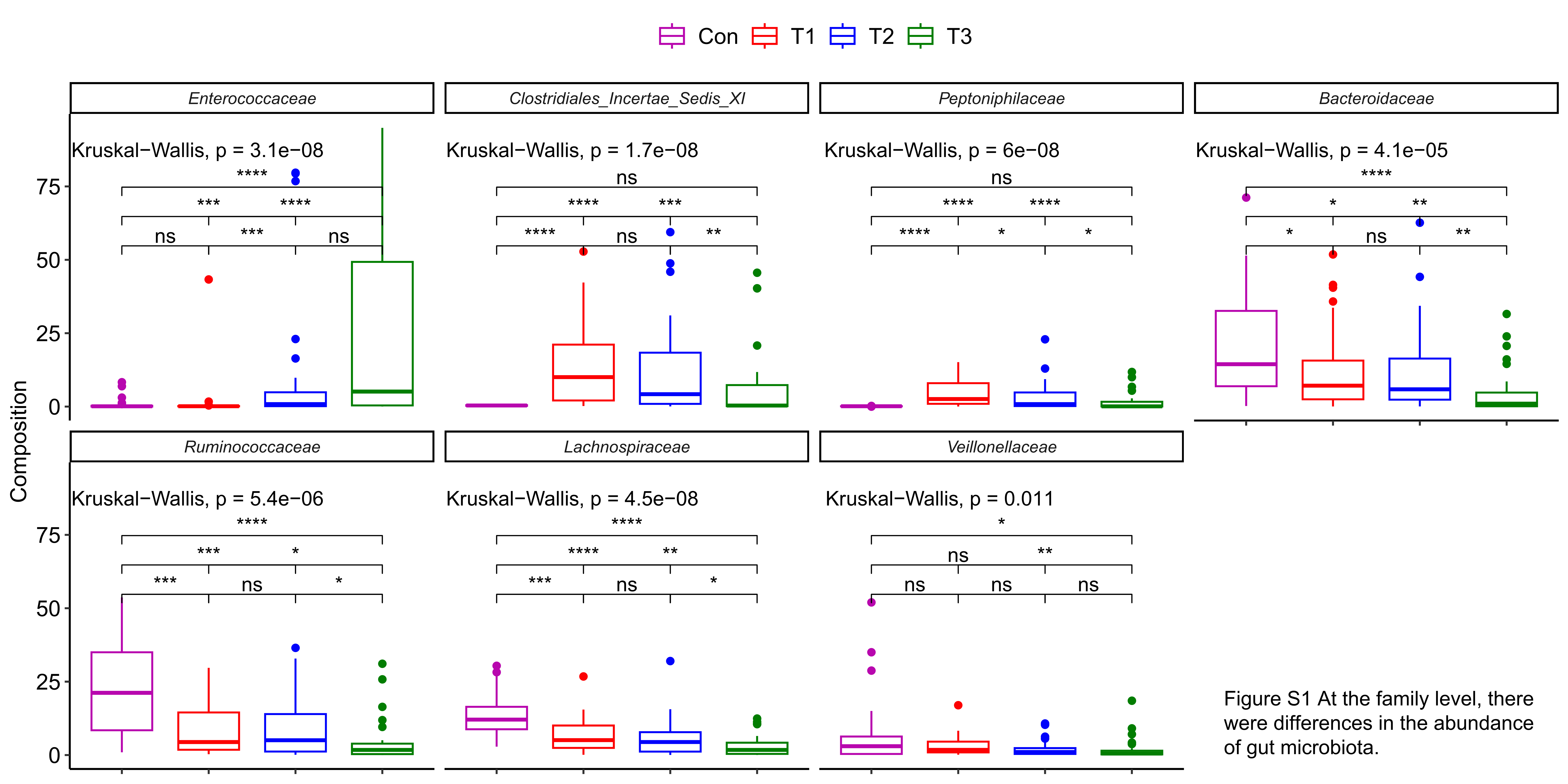

Supplement: Fig. S1 — Differences of gut microbiota. [file spectrum.01178-24-s0001.tif]
